# Editorial: Systems Biology and Omics Approaches for Understanding Complex Disease Biology

**DOI:** 10.3389/fgene.2022.896818

**Published:** 2022-04-12

**Authors:** Amit Kumar Yadav, Sanjay Kumar Banerjee, Bhabatosh Das, Kumardeep Chaudhary

**Affiliations:** ^1^ Translational Health Science and Technology Institute, NCR Biotech Science Cluster, Faridabad, India; ^2^ Department of Biotechnology, National Institute of Pharmaceutical Education and Research (NIPER), Guwahati, India; ^3^ Charles Bronfman Institute for Personalized Medicine, Icahn School of Medicine at Mount Sinai, New York, NY, United States

**Keywords:** multi-omics, systems biology, transcriptomics, proteomics, metabolomics, network biology, disease biology, machine learning

High-throughput omics technologies have seamlessly galvanized the fields of big data and systems biology (Karczewski and Snyder, 2018). The amalgamation of omics techniques (genomics, transcriptomics, proteomics, metabolomics, and lipidomics etc.) and computational methods have enhanced our understanding of diseases in exquisite molecular detail (Adela et al., 2019; Aggarwal et al., 2020). Since computational methods help to unlock the potential of big-data (Shilo et al., 2020; Subramanian et al., 2020; Tolani et al., 2021), we solicited articles that applied systems biology approaches to complex diseases. The hosted topic received an excellent response and 13 manuscripts were accepted after careful editing.

Few studies harnessed the power of publicly available transcriptomic datasets. Khatri et al. studied 19 transcriptomics datasets to understand pancreatic ductal adenocarcinoma (PDAC). They constructed a support vector machine (SVM) classification model to predict a 9-gene biomarker panel of secretory proteins capable of predicting disease outcomes and patient risk stratification. Kowalski et al. evaluated the expression of epigenetics-related genes after valproic acid, carbamazepine, or phenytoin exposure in fetal development. Using weighted gene co-expression network analysis (WGCNA) on transcriptome data, they identified genes that correlated with Fetal Valproate Syndrome, and Fetal Hydantoin Syndrome.

Some studies applied proteomics or metabolomics analysis to study complex diseases. Using quantitative proteomics (iTRAQ), Das et al. studied the slow recovery in zebrafish males compared to females, following hypoxic-ischemic insult. The analysis exposed a sex-based difference in the neuronal cell recovery, with the increased levels of H3K9me3 in males confirmed through ChIP-qPCR. This can be used to develop novel targets for gender-specific therapeutic strategy. Another proteomics study by Zhou et al. used tandem mass tag (TMT) proteomics to understand the connection between “Heart failure with preserved ejection fraction” (HFpEF) and hypertension (HTN). The functional and network analysis revealed seven common differentially expressed proteins in HFpEF and HTN, for which molecular docking studies were performed to identify therapeutic targets. Sardar et al. integrated proteomics and clinical data to identify biomarkers of COVID-19 progression using artificial intelligence. Using feature selection and cross-validation on normalized protein expression data, a LogitBoost model was developed. They also identified 18 potential proteins for drug repurposing, when understanding of COVID-19 disease was in its early stages (Chatterjee et al., 2020). The prominent clinical abnormalities also included cardiovascular functions (Shen et al., 2020), which was also studied recently (Rizvi et al., 2022). The authors developed CovidPrognosis webserver for predicting patient survival, for assisting in rapid patient triaging. Wang et al. studied the serum metabolome of 99 patients with acute ischemic stroke to identify biomarkers and its heterogeneous subtypes. Using PCA and OPLS-DA analysis, the authors identified 18 metabolites including oleic acid, linoleic acid, arachidonic acid, which could differentiate between stroke patients and healthy individuals. The authors also identified differences in ischemic stroke subtypes to explore pathophysiological mechanisms.

Integrating multiple omics data was another popular theme for some articles. Zhao et al. presented an interesting study on the pathogenesis of polycystic ovary syndrome (PCOS)- the most common, endocrine and metabolic disease in women of reproductive age. Limited studies have been performed with multiomics analyses of granulosa cells (GCs) considering epigenetics as a regulatory factor. The authors systematically investigated the differences in the mRNA-miRNA-lncRNA transcriptome and genome-wide DNA methylation modification profiles and their regulatory networks. The data revealed that all differentially expressed genes were associated with steroid biosynthesis and glycolysis/gluconeogenesis pathways. Diabetic retinopathy (DR) requires early diagnostic markers and effective treatment strategies. Another interesting integrative computational approach by Kumari et al. was devised to capture differentially expressed genes that were also the targets of miRNA, as well as depicted atypical methylation patterns. The authors identified hub genes and network modules from the PPIs of the early and late disease genes. They also identified the pathways related to oxidoreductase activity, extracellular matrix binding, immune response, cell adhesion, PI3K-Akt signaling pathway, ECM receptor interaction and leukocyte migration. They reported 7 hub genes and 9 early genes as potential candidates for prognostic, diagnostic, or therapeutic application. A fascinating approach to integrate metabolism with the regulatory-metabolic network using transcriptomics data was demonstrated by Sun et al. to understand tumour heterogeneity in hepatocellular carcinoma (HCC). The authors studied disease perturbation in regulation and metabolism using unified mechanistic modeling approach, which used transcriptomics data with regulatory-metabolic network model to understand HCC stratification. They identified transcription factors and target genes impacting tumorigenesis and integrated this information with constraint-based models identifying five important genes associated with cancer growth. Non-negative matrix factorization was used for stratification of differential genes from TCGA samples to understand HCC pathways and find potential targets. In another excellent multiomics approach, the pathogenesis of carotid atherosclerosis (CAS) (a cause of stroke) was studied by Ji et al. with respect to the interactions between gut microbiome and metabolome. Authors attempted an integrated analysis of the transcriptome (from GEO) with in-house generated metabolome and microbiome data for in-depth understanding of the “microbiota–metabolite–gene” axis in the pathogenesis of CAS. Interestingly, the study identified α-N-Phenylacetyl-L-glutamine as an increased metabolite in CAS patients. *FABP4* was the most upregulated gene and was positively associated with *Acidaminococcus*, an anaerobic bacteria living in the human gut. The authors integrated and overlaid different omics data to understand CAS pathogenesis. However, the study could have benefitted more from generating transcriptome data from the same patients as the microbiome and metabolome data.

Biswas et al. developed a sophisticated analysis platform-ADOPHIN, to allow the analysis of pan-omics data in context of the Human interactome. They developed a meta-interactome network with protein-protein interactions (PPIs), regulatory interactions between miRNAs and their respective target genes, transcription factors and their targets. The authors discovered topologically important nodes (TINs) with regulatory networks between various biomolecules (proteins, transcription factors, or miRNAs), linked to signaling and metabolic pathways. The genes, proteins or miRNA from multi-omics data are mapped onto the compiled interactome to capture the biological context-specific interactions, as demonstrated by authors in cervical, breast and ovarian cancers. Such meta-interactome mining approaches with cross-pathway links and connectivity analysis, provide a user-friendly method to explore multi-omics data.

Though excellent studies in their own right, some of the studies may require, and even benefit from independent validation. Furthermore, the power of such integrated analysis can increase with more data types, beyond methylation and transcription/gene expression (Hasin et al., 2017; Yan et al., 2018). This can help in triaging more functional interconnections and discovery of relevant candidates for further research.

Apart from exceptional articles, the topic also had two excellent reviews. The review by Zamith-Miranda et al. appraises the biogenesis, composition and functions of fungal extracellular vesicles using multi-omics studies. Shedding of extracellular vesicles is a conserved process across all three kingdoms of life. The mechanisms and sites of fungal extracellular vesicle formation, their nucleic acid content and importance in virulence, pathogenicity and antimicrobial resistance are discussed. The review concludes with the current knowledge gaps in the extracellular vesicles biology and their future. An excellent review on the current landscape of Single-Cell Transcriptomics (scRNA-seq) data acquisition and bioinformatics analysis is presented by Adil et al. scRNA-seq has emerged as an instrumental technique to decipher cellular heterogeneity in complex diseases. However, the volume, granularity and sparsity of data poses outstanding challenges-in data generation and downstream analysis. An overview of scRNA-seq profiling techniques and biophysical cell-isolation methods is followed by the widely-used tools for sequencing read-alignment and mRNA expression quantification. The bottlenecks and current software solutions reviewing the methods for normalization, batch-effect removal, imputation, dimensionality reduction, subtype/cluster identification are also covered. Finally, the review discusses the multiple evolving strategies to integrate multi-omics datasets at the single-cell level.

The topic has covered multiple omics with different computational methods, network analysis and modeling to study a diverse array of biological problems in complex diseases. We hope this interesting assortment of article collection invigorates the readers towards novel applications of multiomics for deeper dissection of disease biology.
